# Assessment of Muscle Synergies in Chronic Ankle Instability Patients During Unanticipated and Anticipated Landing

**DOI:** 10.3390/bioengineering11121237

**Published:** 2024-12-06

**Authors:** Zhifeng Zhou, Datao Xu, Meizi Wang, Tianle Jie, Julien S. Baker, Huiyu Zhou, Yaodong Gu

**Affiliations:** 1 Faculty of Sports Science, Ningbo University, Ningbo 315211, China; zhouzhifeng_nbu@outlook.com (Z.Z.); xudatao3@gmail.com (D.X.); meiziwang@polyu.edu.hk (M.W.); jietianle_nbu@outlook.com (T.J.); jsbaker@hkbu.edu.hk (J.S.B.); zhouhuiyu@nbu.edu.cn (H.Z.); 2 Department of Biomedical Engineering, Faculty of Engineering, The Hong Kong Polytechnic University, Kowloon, Hong Kong SAR 999077, China; 3 Faculty of Engineering, University of Szeged, 6720 Szeged, Hungary

**Keywords:** neuromuscular activation, muscle synergy, non-negative matrix factorization, unanticipated landing, chronic ankle instability

## Abstract

Ankle sprains are a common injury among athletes and the general population, with chronic ankle instability (CAI) being a frequent complication. CAI patients often display altered neuromuscular control adaptations. This study analyzed muscle synergy patterns in 20 CAI patients during anticipated and unanticipated landing tasks to understand their neuromuscular adaptation strategies. Using Nesterov non-negative matrix factorization and K-means clustering, the study identified distinct muscle activation patterns. Results indicated that during unanticipated landings, the gluteus maximus and vastus lateralis showed increased activation weight, while the medial gastrocnemius was more active in anticipated landings. This study highlights that CAI patients display unique muscle synergy patterns during unanticipated landings, relying more on proximal muscles such as the gluteus maximus and vastus lateralis. This adaptation reflects the proximal muscle strategy to enhance stability and compensate for impaired ankle function in unpredictable situations.

## 1. Introduction

Ankle injuries, particularly ankle sprains, are common in athletes and the general population during everyday activities. These injuries not only cause acute pain and functional impairment but can also lead to chronic issues, such as chronic ankle instability (CAI) [1]. CAI is characterized by recurring ankle sprains and persistent feelings of instability following an initial ankle sprain. Epidemiological studies indicate that approximately 40% to 70% of individuals with an ankle sprain may develop CAI. CAI not only impacts athletic performance but also increases the risk of future injuries and can result in long-term functional impairments [2]. The complexity of ankle injuries lies in their involvement of multiple factors beyond a single structural issue. This includes proprioceptive loss, altered neuromuscular control, and coordination of motor skills. Ankle instability is often associated with a loss of proprioception and changes in neuromuscular control strategies, with these factors collectively affecting joint stability and function [3].

In studying the movement strategies of chronic ankle instability (CAI) patients, unanticipated cutting tasks are a critical focus. These tasks often lead to marked alterations in movement strategies, particularly when comparing anticipated versus unanticipated conditions. Unanticipated tasks place greater demands on performance, requiring increased muscle activation and more complex movement control strategies [4]. Such tasks typically require athletes to respond rapidly in uncertain environments, challenging their dynamic stability and motor coordination [5]. Studies indicate that CAI patients often display different movement strategies compared to healthy individuals during unanticipated cutting tasks. For example, Kim’s research found that CAI patients rely more heavily on tibialis anterior activation during both the early and late stages of landing and cutting tasks [6]. This adjustment serves as a compensatory strategy for the inadequacies of ankle joint function. Moreover, unanticipated landing tasks not only reveal deficiencies in CAI patients’ movement control but also highlight their adaptability in managing sudden situations [7]. These adaptations may involve cognitive processing of movement tasks, adjustments in neuromuscular control strategies, and responses to unstable environments [8]. Compared to healthy individuals, CAI patients may show greater movement instability and slower adaptability during unanticipated tasks, potentially increasing their risk of injury in real-world activities. Specifically, these patients tend to rely more on proximal muscles, such as the gluteus maximus and vastus lateralis, to maintain overall stability during landings [9]. Ankle injuries lead to adaptive changes in the central nervous system, which affect motor control. Consequently, proximal joints of the injured lower limb may exhibit neuromuscular control deficits. Thus, investigating movement patterns during unanticipated landing tasks is vital to creating efficient rehabilitation strategies and interventions [10].

Muscle synergy patterns are a central concept in the study of neuromuscular control and play a crucial role in motor control research. Initially proposed by Bernstein, muscle synergies refer to groups of “cooperating” muscle groups. This concept has evolved significantly, especially with advancements in non-negative matrix factorization (NNMF) algorithms [11]. NNMF simplifies complex multidimensional data by decomposing muscle activation signals into several time-invariant synergy modules, allowing for detailed analysis through specific task activation coefficients [12]. Building upon this process, the NNMF algorithm identifies modules associated with time-invariant muscle synergy patterns from the input muscle activation matrix and generates activation coefficients for individual tasks [13]. This decomposition method enables researchers to identify and quantify the synergistic actions of muscle groups during movement, revealing their functions in different motor tasks. The advantage of this technique lies in its non-negativity constraint on the original data, which preserves the true characteristics of the data while accurately capturing the core patterns of muscle activity [14,15]. In CAI research, NNMF algorithms have been widely applied. Studies have shown that NNMF can provide a detailed analysis of muscle synergy patterns in CAI patients across various motor tasks, helping to identify key muscle groups and their synergies associated with CAI [16,17]. For instance, Ghislieri et al. found that CAI patients exhibited poorer balance in single-leg tasks compared to healthy controls and demonstrated reduced muscle synergy in the absence of visual information. This indicates a high reliance on visual input to compensate for proprioceptive deficits affecting the ankle joint [18].

Therefore, this study aims to assess and compare muscle synergy patterns in patients with CAI during anticipated and unanticipated landing tasks. We hypothesize that CAI patients will exhibit significant changes in their muscle-specific functional roles during unanticipated landings, which will better reflect their control strategies when faced with unexpected situations compared to anticipated landings. By analyzing the muscle activity of CAI patients in these two different contexts, we hope to reveal the neuromuscular coordination capabilities and adaptive responses required for effective movement execution. Furthermore, the findings of this research will provide important theoretical support for understanding CAI patients’ ability to maintain stability and balance in dynamic environments, thereby guiding clinical rehabilitation interventions to improve patients’ athletic performance and quality of life.

## 2. Materials and Methods

### 2.1. Participant

In this study, 20 participants with chronic ankle instability (CAI) were recruited. Each participant completed the self-administered Cumberland Ankle Instability Tool (CAIT) questionnaire, which gathered information on anthropometric parameters, the frequency of lateral ankle sprains, and their history of medical consultations. According to the criteria set by the International Ankle Consortium, CAI is defined by the following: (1) a CAIT score of 24 or lower, (2) a history of traumatic ankle sprains requiring medical attention on two or more occasions, and (3) recurrent lateral ankle sprains lasting for at least 6 months or occurring frequently. To account for the potential impact of limb dominance on the experiment, all participants’ right legs were designated as the dominant limb, defined as the preferred leg for kicking a ball. Participants were fully briefed on the study’s objectives, requirements, and procedures, and provided their written informed consent. The study protocol was approved by the Research Ethics Committee of Ningbo University (Approval Number: RAGH20240622).

### 2.2. Experimental Protocol and Data Recordings

**Figure 1:**
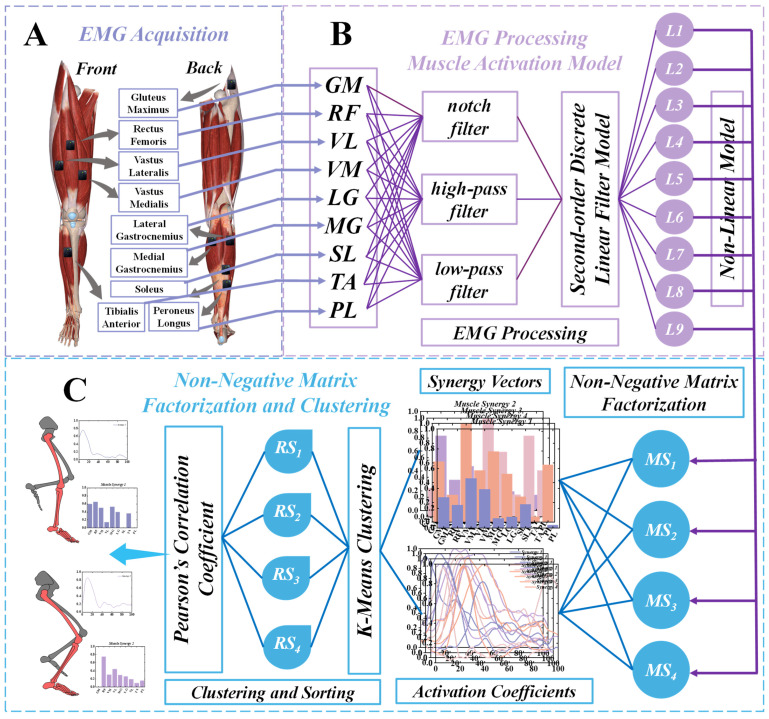
The overall workflow of the current study (**A**) Acquisition of sEMG. (**B**) Detailed Processing for sEMG data. (**C**) Muscle synergy extraction using the NNMF algorithm and Sorting of muscle synergies using the K-means clustering algorithm.

This study employed a Kistler force platform alongside an eight-camera Vicon motion capture system from Oxford Metrics Ltd. (Oxford, UK) to simultaneously capture dynamic and kinematic data. Kinematic data were acquired at a frequency of 200 Hz, while dynamic data were collected at 1000 Hz. Mark points were set according to the 2392 generic model, and EMG sensors were positioned following SENIAM guidelines [19]. To prepare the measurement area, hair was shaved from the skin and the area was wiped with alcohol to minimize the impedance at the skin-electrode interface. Eight EMG sensors (Delsys, Boston, MA, USA) were placed on the muscle belly of the soleus (SL), medial gastrocnemius (MG), lateral gastrocnemius (LG), tibialis anterior (TA), peroneus longus (PL), rectus femoris (RF), vastus medialis (VM), and vastus lateralis (VL) to quantify muscle activation (Figure 1A).

Before the formal experiment, each movement was preceded by adequate warm-up exercises. During the warm-up phase, participants first familiarized themselves with the task procedure and landed several times from a two-step platform to ensure they had mastered the correct landing techniques. In the subsequent unanticipated landing task, participants were required to descend from two steps, each 20 cm high, and land on a landing board with their right leg. They were required to remain on the force platform until their foot touched the ground. Participants were unaware of whether the springboard was tilted. A sponge was placed under the springboard; and when the foot made contact, the sponge collapsed, causing the board to tilt. All tasks were performed barefoot, and each participant was required to complete 5 trials of the unanticipated and anticipated landing task successfully. The order of the anticipated and unanticipated landing tasks was alternated in a randomized sequence to simulate unexpected conditions and ensure the non-prepared nature of the unanticipated landings. For analysis purposes, the results were divided into ‘anticipated’ and ‘unanticipated’ groups based on the specific task condition performed by the participants during the trial. The unanticipated trial was considered successful if the entire foot contacted the ground, and the trial was deemed unsuccessful if the participant could not maintain balance. Only trials that were considered successful were included in the analysis.

### 2.3. Data Processing

This study utilized Vicon Nexus 2.14.0 software (Vicon Metrics Ltd., version 2.14.0, Oxford, UK) to identify and acquire three-dimensional marker trajectories and ground reaction force (GRF) data, which were exported in C3D format. Concurrently, a custom MATLAB 2022b (MathWorks, Inc., Natick, MA, USA) was employed to construct a muscle activation model from the raw surface electromyography (EMG) signals. Data analysis focused on the posture phases of each task. For the landing task, a GRF threshold of 10 N was established to define the posture phase. This threshold was set to accurately capture the stability and mechanical characteristics during the landing process. The EMG data underwent a detailed processing sequence. Initially, a low-pass filter with a cutoff frequency of 450 Hz was applied to remove high-frequency noise and interference, while a high-pass filter with a cutoff frequency of 20 Hz was used to eliminate low-frequency drift and artifacts. This preliminary filtering step aimed to clean the signal and retain meaningful electromyographic information. Following filtering, the EMG data were rectified, converting all negative values to positive. This step facilitates subsequent analysis by ensuring that the signal is uniformly positive. The rectified data were then smoothed using a low-pass filter with a cutoff frequency of 10 Hz, which reduced high-frequency fluctuations and produced a more stable signal suitable for analysis. Finally, the smoothed EMG signals were normalized using peak normalization [20]. This process standardized each signal by scaling the maximum value to a common reference, thus removing individual differences between trials or participants and enabling accurate comparisons under different conditions (Figure 1B).

**Figure 2:**
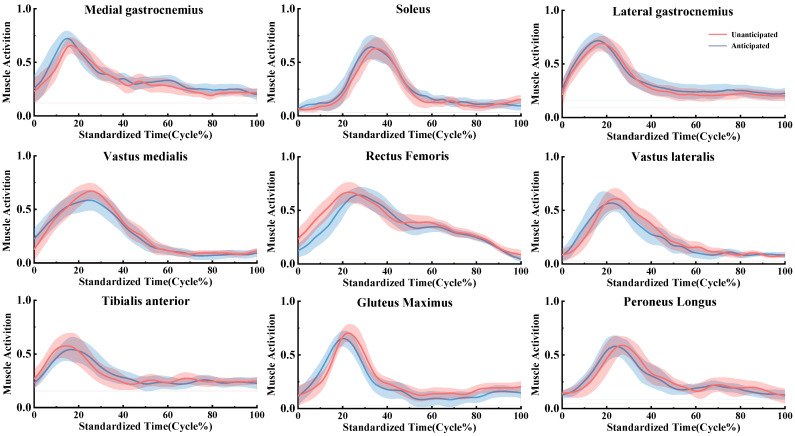
Muscle activation profiles during landing in the Unanticipated and Anticipated groups.

Through these meticulous processing steps, we obtained normalized EMG signals that provided a reliable foundation for further analysis of muscle activation patterns. This approach ensured a thorough examination of the posture phases, offering insights into the relationship between muscle activation and movement performance (Figure 2).

### 2.4. Nesterov Non-Negative Matrix Factorization Extracts Muscle Synergies

Non-negative matrix factorization (NNMF) is a method that exploits the intrinsic relationships within data to derive a mapping matrix. By projecting high-dimensional data onto a lower-dimensional subspace, NNMF is an effective tool for data clustering and dimensionality reduction tasks (Figure 1C). Specifically, NNMF decomposes high-dimensional data into the product of two lower-dimensional matrices. However, NNMF encounters certain efficiency issues during the decomposition process. Therefore, this paper adopts a novel approach, NeNMF, to decompose electromyography (EMG) signals. By utilizing NeNMF, we aim to enhance the efficiency and effectiveness of the decomposition, allowing for better representation of the underlying patterns in the EMG data and facilitating improved analysis and interpretation. The EMG data matrix $V_{mn}$ is of dimensions m×n, where m denotes the number of muscles and n denotes the number of data points [21]. The underlying principle of NeNMF is described as follows:

(1) $$V_{mn}\approx (WH{)}_{mn}=\sum_{i=1}^{k}\;W_{mi}H_{in}=V_{mn}^{\prime }$$

To approximate the decomposition V≈WH, we first need to define a cost function to quantify this approximation [22]. According to Equation (1), and starting from an initial $W^{1}\geq 0$, the block coordinate descent method iteratively alternates between solving the subproblems defined in the optimization framework, the function can be defined as follows:

(2) $$H^{t+1}=arg\;\underset{H\geq 0}{min}\;F(W^{t},H)=\frac{1}{2}∥X-W^{t}H{∥}_{F}^{2}$$

After optimization, we obtain the following:

(3) $$W^{t+1}=arg\;\underset{W\geq 0}{min}\;F(W,H^{t+1})=\frac{1}{2}{∥X^{T}-H^{t+1}W^{T}∥}_{F}^{2}$$

This study uses Nesterov’s Optimal Gradient Method (OGM) to optimize the NNMF process. By applying Nesterov’s approach, we aim to increase both the speed and accuracy of convergence, leading to more efficient data analysis, such as better feature extraction and representation.

In the realm of optimization, recent findings indicate that smooth optimization methods based on gradients can lead to improved convergence rates. Because the gradient satisfies Lipschitz continuity, we can effectively apply Nesterov’s method to optimize Equations (2) and (3). To facilitate this process, we define two sequences that are updated during each iteration, and these sequences are described as follows:

(4) $$\begin{array}{cc} H_{k} & =\arg\;\underset{H\geq 0}{min}\;\phi (Y_{k},H)=F\left(W^{t},Y_{k}\right)\\ & +\left\langle \nabla_{H}F(W^{t},Y_{k}),II-Y_{k}\right\rangle +\frac{L}{2}∥II-Y_{k}{∥}_{F}^{2} \end{array}$$

And

(5) $$Y_{k+1}=II_{k}+\frac{\alpha_{k}-1}{\alpha_{k+1}}(II_{k}-II_{k-1})$$

Previous research indicates that the optimized NeNMF method offers significant advantages over NNMF solvers. Specifically, NeNMF maintains a comparable computational complexity while achieving faster convergence rates in each iteration. This efficiency allows for quicker results, making it particularly beneficial for applications that require timely matrix decomposition. Additionally, the optimized NeNMF effectively captures the underlying structures in the data, ensuring accurate representation and interpretation.

Additionally, the Variance Accounted For (VAF) value indicates the extent to which the global muscle activity (${VAF}_{global}$) and the local muscle activity (${VAF}_{muscle}$) in the electromyography (EMG) data can be explained by their respective number of synergies:

(6) $${VAF}_{global}=1-\frac{\sum_{m,n}\;(V-V_{r}{)}_{m,n}^{2}}{\sum_{m,n}\;V_{m,n}^{2}}$$

(7) $${VAF}_{muscle}=1-\frac{\sum_{n}\;(V-V_{r}{)}_{m_{each}n}^{2}}{\sum_{n}\;V_{m_{each}n}^{2}}$$

At the same time, the number of synergies must meet the following criteria: the global muscle variance accounted for (${VAF}_{global}$) should be ≥90%, and the local muscle variance accounted for (${VAF}_{muscle}$) should be ≥75% [23].

### 2.5. K-Means Clustering Algorithm for Sorting Muscle Synergies

In this study, we employed the K-means clustering algorithm to classify muscle synergies. First, we organized the muscle activity data into a matrix X. After standardizing the data, we applied the K-means algorithm to identify different muscle synergy patterns [24]. The initial cluster centers $\mu_{i}$ were chosen randomly and optimized through the following steps:

(8) $$\mathrm{assign}\left(x_{j}\right)=\arg\;\underset{i}{min}\;∥x_{j}-\mu_{i}{∥}^{2}$$

Each cluster center $\mu_{i}$ to be the mean of all data points in the cluster:

(9) $$\mu_{i}=\frac{1}{\left|C_{i}\right|}\sum_{x\in C_{i}}\;x$$

The assignment and update steps continue until the change in cluster centers is less than a specified threshold ϵ or until the maximum number of iterations, max_iters is reached. The change in cluster centers is calculated as follows:

(10) $$\Delta \mu_{i}=∥\mu_{i}^{new}-\mu_{i}^{old}∥$$

After completing the K-means clustering, we further refined the classification of each subject’s synergy vectors by calculating the Pearson correlation coefficient $r_{ij}$ between each synergy vector $W_{j}$ and each cluster center $\mu_{i\;}$ [25]. The Pearson correlation coefficient is computed as:

(11) $$r_{ij}=\frac{Cov(W_{j},\mu_{i})}{\sigma_{W_{j}}\sigma_{\mu_{i}}}$$

where $Cov(W_{j},\mu_{i})$ is the covariance between the synergy vector and the cluster center, and $\sigma_{W_{j}}$ and $\sigma_{\mu_{i}}$ are their respective standard deviations. If $r_{ij}$ is greater than 0.6, we consider the synergy vector $W_{j}$ to be highly similar to the cluster center $\mu_{i}$ and assign $W_{j}$ to cluster i. Once the synergy vectors have been classified, their corresponding activation coefficients C are automatically grouped into the same category [26]. This method, combining K-means clustering with Pearson correlation, provides a refined classification framework that helps in analyzing different muscle synergy patterns and their variations under different conditions.

### 2.6. Statistical Analysis

The study commenced using statistical analysis employing the Shapiro–Wilk test to evaluate the normality of the data. If the data met the criteria for normal distribution, we proceeded with a paired *t*-test to analyze the differences between the two groups [27]. This approach allows us to discern whether there are substantial differences between the means of the two related samples, thereby shedding light on any significant effects or differences between the groups [28]. The formula for the paired *t*-test is given by:

(12) $$t=\frac{\bar{d}-\mu_{0}}{\frac{s_{d}}{\sqrt{n}}}$$

In which,

(13) $$\bar{d}=\frac{\sum_{i=1}^{n}\;d_{i}}{n}$$

In the paired t-test, this refers to the mean of the differences between paired samples:

(14) $$s_{d}=\sqrt{\frac{\sum_{i=1}^{n}\;{\left(d_{i}-\overset{¯}{d}\right)}^{2}}{n-1}}$$

It is the standard deviation of the differences between paired samples, and n represents the number of paired samples.

To study the movement patterns of CAI patients’ lower limbs with Statistical Parametric Mapping (SPM), we began by extracting and preparing the dataset. We used a MATLAB script to convert stance phase data into a time series of 101 points. Following this, we conducted the SPM analysis by applying thresholds derived from random field theory and creating the necessary SPM curves [29]. If SPM values go beyond the critical thresholds, it shows that there are significant differences in activation coefficients between the groups at that specific time, revealing important variations in muscle activity [30]. This method helps identify statistically significant differences within the time series, allowing for a more accurate analysis of the movement patterns in CAI patients.

## 3. Results

### 3.1. Muscle Synergy Number via NeNNMF Decomposition

**Figure 3:**
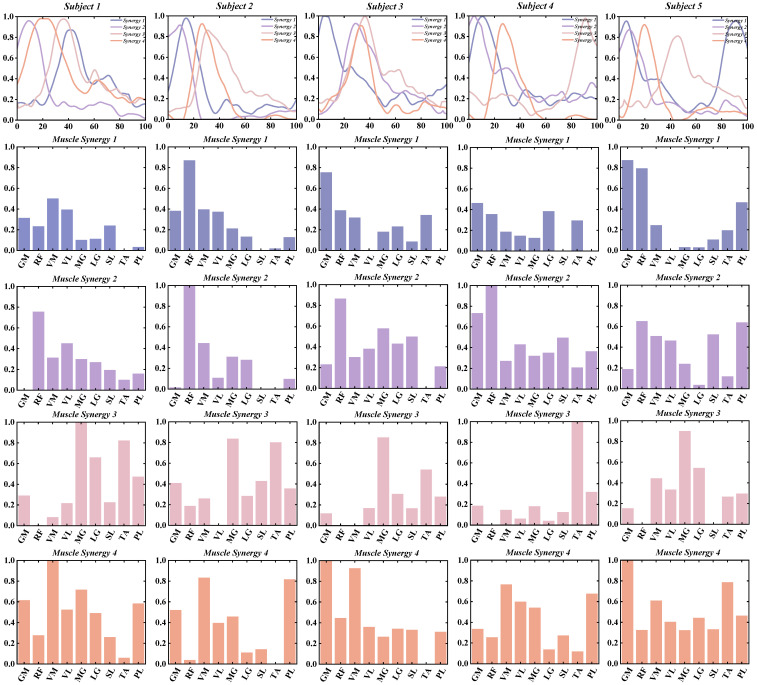
Detailed Presentation of activation coefficient results and synergy vectors for Subjects 1–5 in the Unanticipated Landing Group.

**Figure 4:**
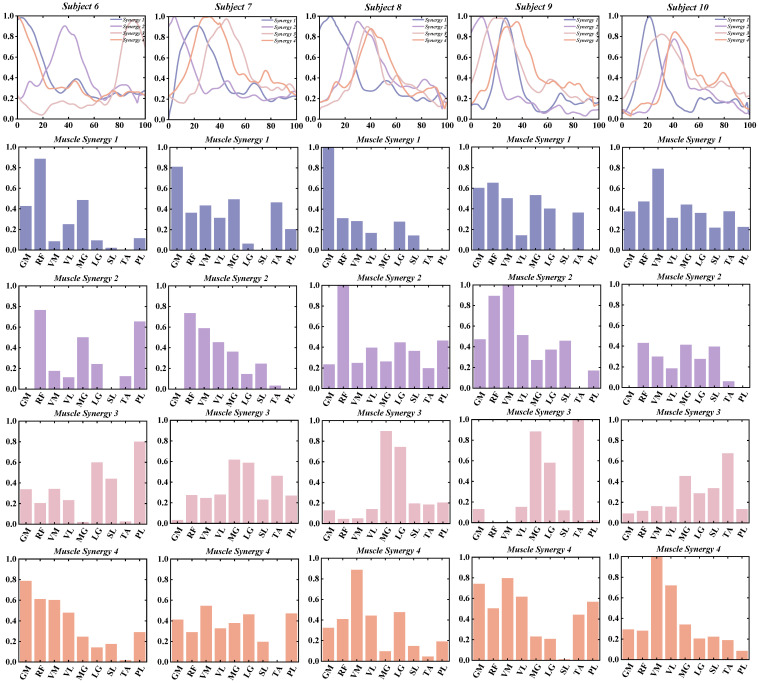
Detailed Presentation of activation coefficient results and synergy vectors for Subjects 6–10 in the Unanticipated Landing Group.

**Figure 5:**
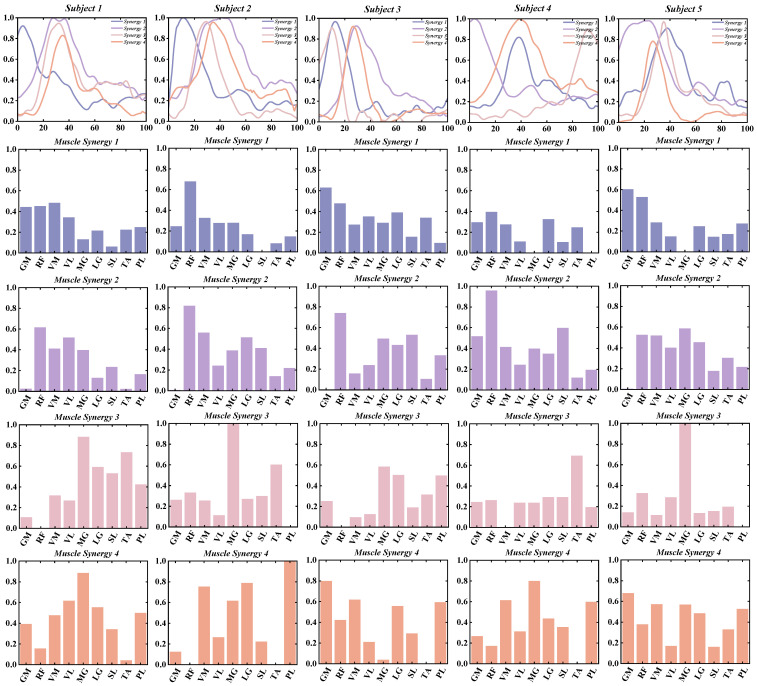
Detailed Presentation of activation coefficient results and synergy vectors for Subjects 1–5 in the Anticipated Landing Group.

**Figure 6:**
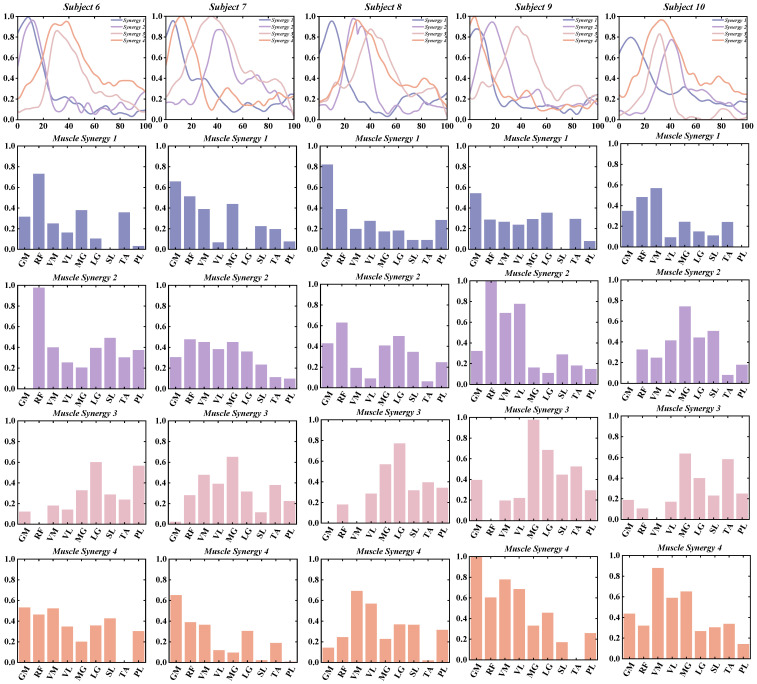
Detailed Presentation of activation coefficient results and synergy vectors for Subjects 6–10 in the Anticipated Landing Group.

This study investigates the muscle synergy patterns in patients with chronic ankle instability (CAI) during unanticipated and anticipated landing tasks. The analysis reveals that most CAI patients exhibit four primary muscle synergy patterns under both landing conditions. These patterns highlight the coordination and variability of muscle interactions in CAI patients across different landing scenarios. To improve clarity in our reporting, we focus exclusively on presenting the coordination vector matrices and activation coefficient matrices for the four primary muscle synergy patterns extracted from each subject group (Figure 3, Figure 4, Figure 5 and Figure 6). These matrices visually represent the muscle synergies under various landing conditions. Specifically, the coordination vector matrices illustrate the relationships between different muscles, while the activation coefficient matrices reflect the intensity and patterns of muscle activation during the landing process.

**Figure 7:**
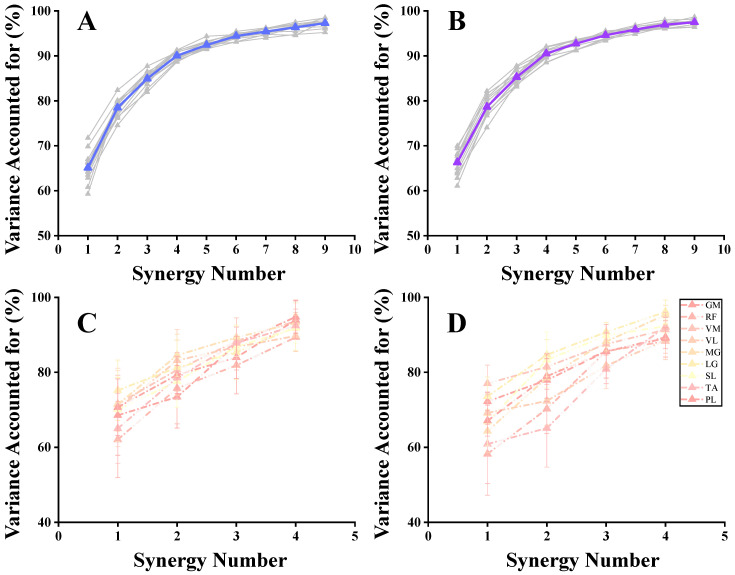
The global and local VAF correspond to each synergy in the Unanticipated and Anticipated groups. (**A**) The global VAF of the unanticipated group; (**B**) The global VAF of the anticipated group; (**C**) The local VAF of the unanticipated group; (**D**) The local VAF of the anticipated group. The gray line illustrates the VAF of each subject, the blue line illustrates the VAF of the unanticipated group and the purple line illustrates the VAF of the anticipated group.

Based on the results from the Variance Accounted For (VAF) analysis, we found that patients with chronic ankle instability (CAI) demonstrated high levels of global VAF all muscle synergy patterns during both unanticipated and anticipated landing tasks (Figure 7).

In the unanticipated landing group, the global VAF values were as follows: 84.9% ± 1.5% with three muscle synergies, 90.0% ± 0.9% with four muscle synergies, and 92.4% ± 0.8% with five muscle synergies. Similarly, in the anticipated landing group, the global VAF values were: 85.3% ± 1.5% with three muscle synergies, 90.5% ± 1.2% with four muscle synergies, and 92.7% ± 0.9% with five muscle synergies (Table 1).

### 3.2. Similarity of Muscle Synergies

**Figure 8:**
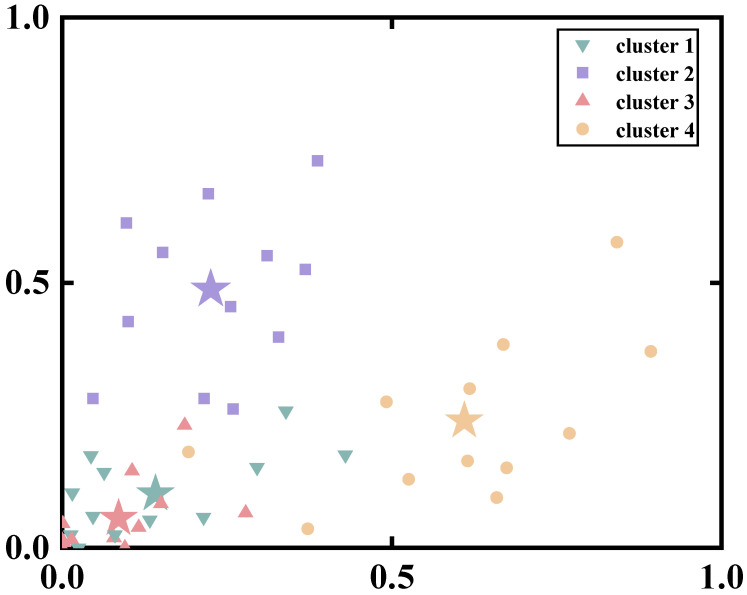
Visualizations of K-means clustering results for all synergy vectors in the Anticipated group. The asterisk represents the cluster center.

Using the Silhouette coefficient to assess the clustering quality, we identified that the clustering effect was maximized when i = 4. This indicates that the optimal number of clusters was four, leading us to use three clustering centers for the reference muscle synergies (Figure 8).

**Figure 9:**
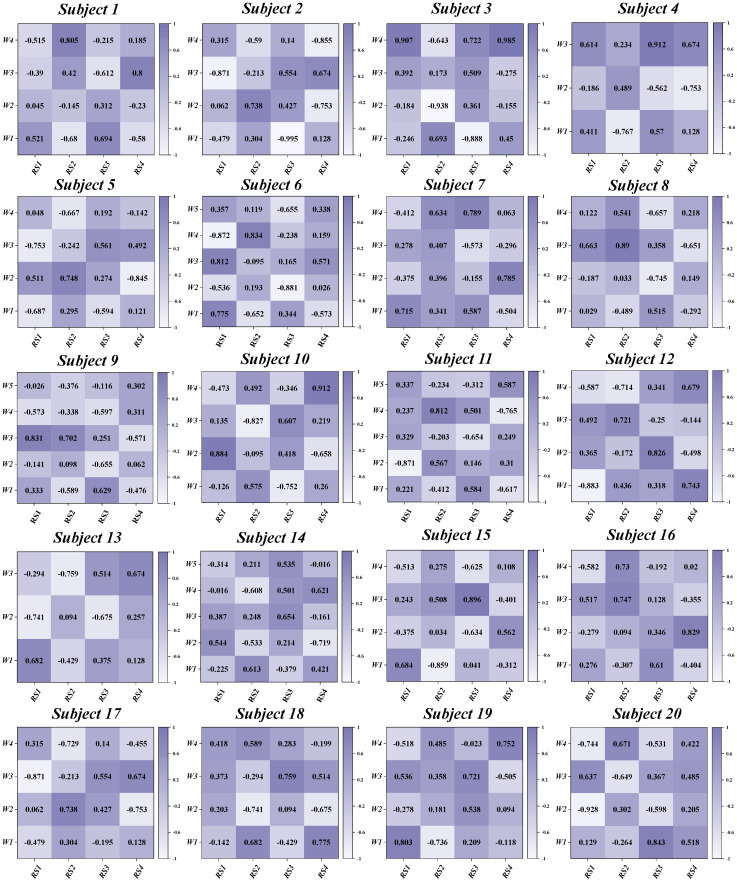
Correlation coefficients between synergy vectors and reference synergies for the 20 participants during the unanticipated landing tasks. W: synergy vectors. RS: Reference Synergy.

**Figure 10:**
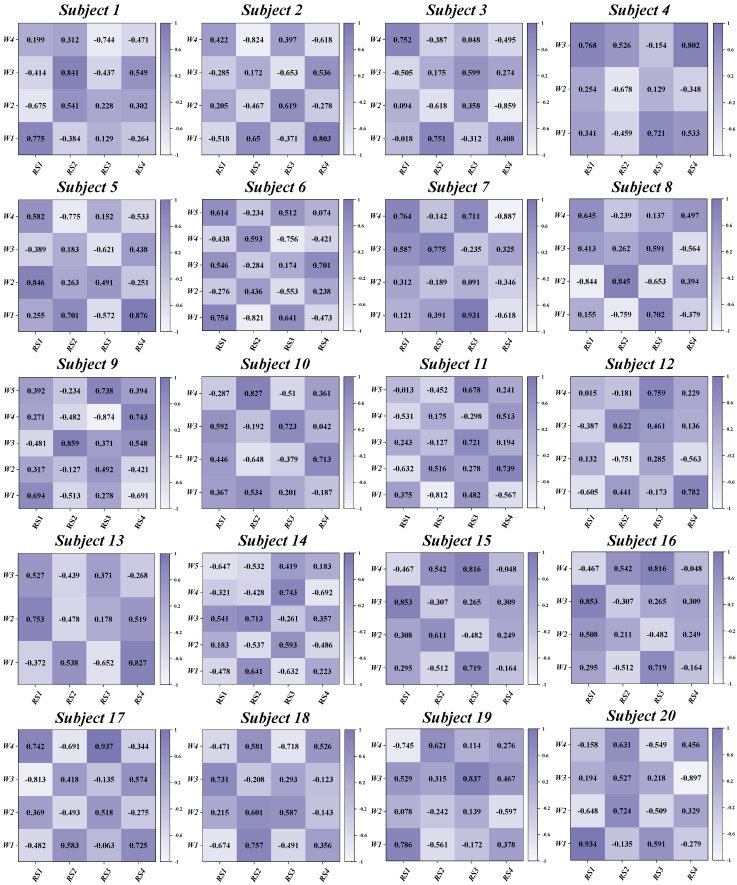
Correlation coefficients between synergy vectors and reference synergies for the 20 participants during the anticipated landing tasks. W: synergy vectors. RS: Reference Synergy.

By using the four cluster centroids as a basis for synergy comparison and applying Pearson correlation coefficients to organize the synergy patterns of individual participants (Figure 9 and Figure 10), we observed that the similarity of reference synergies in the anticipated landing group was significantly higher than in the unanticipated landing group.

### 3.3. The Functional Interpretation of Muscle Synergies

**Figure 11:**
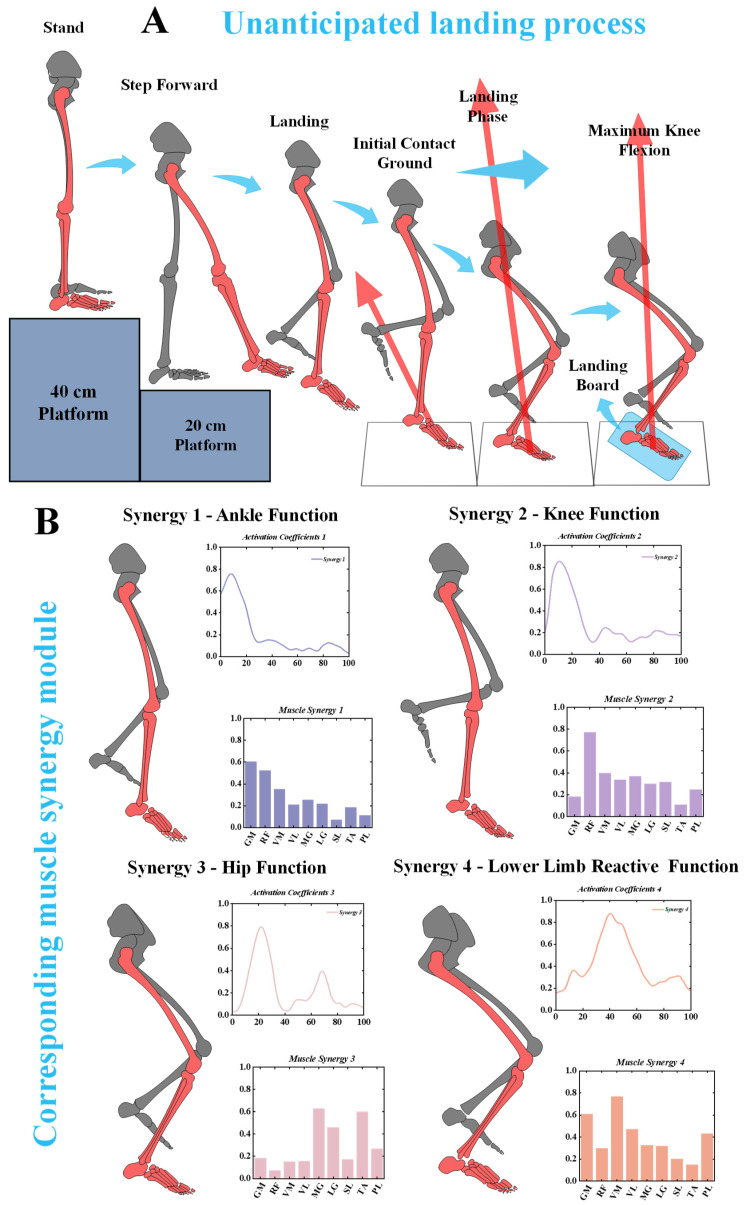
The specific landing biomechanical test and corresponding muscle synergy module. (**A**) Unanticipated landing biomechanical test. (**B**) Corresponding muscle synergy module.

Based on the muscle synergy analysis, we defined muscle weight exceeding 0.3 as indicative of activation. The results revealed distinct activation patterns for different muscle synergies during the landing process (Figure 11A).

In Synergy 1, the activation levels of the gluteus maximus (GM), rectus femoris (RF), and vastus medialis (VM) were relatively high, suggesting that this synergy is closely associated with hip joint function during the landing phase. Synergy 2 displayed high activation levels in the RF, vastus medialis (VM), vastus lateralis (VL), lateral gastrocnemius (LG), medial gastrocnemius (MG), and soleus (SL). This pattern is likely related to the stabilization of the knee joint during landing. Synergy 3 primarily involved the MG, LG, and tibialis anterior (TA), with significant activation occurring during the initial 0–20% of the landing phase. This indicates the critical role of these muscles in controlling the ankle during the initial contact phase. Finally, Synergy 4 showed activation in multiple muscle groups, including the GM, VM, VL, LG, MG, and PL. This pattern highlights that CAI patients rely on the coordinated effort of several muscle groups to maintain stability during the latter part of the landing phase (Figure 11B).

### 3.4. Specific Characteristics of Muscle Synergy

**Figure 12:**
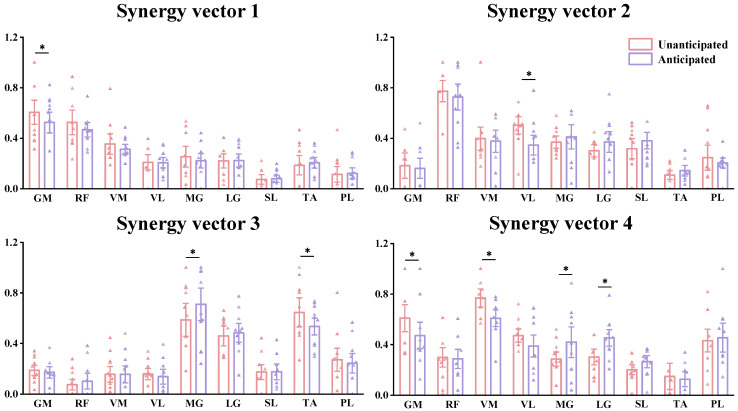
Muscle synergy vectors extracted from the Unanticipated and Anticipated groups. * significant difference with *p* < 0.05.

Based on the results from Muscle Synergy Vectors (Figure 12), in Synergy 1, we observed that the relative weights of most specific muscles did not show significant differences between the Unanticipated and Anticipated groups. The only notable difference was that the relative weight of the GM in the Unanticipated group was significantly higher than that in the anticipated group (*p* = 0.041). In Synergy 2, compared to the Anticipated group, the VL in the Unanticipated group was significantly larger, with a notable difference between the two groups (*p* = 0.043). In Synergy 3, the MG in the Anticipated group was significantly larger than that in the Unanticipated group (*p* < 0.05), while the TA in the Unanticipated group was significantly larger than that in the Anticipated group (*p* < 0.05). In Synergy 4, the GM (*p* < 0.05) and the VM (*p* < 0.001) in the Unanticipated group were significantly greater than those in the Anticipated group. Conversely, the MG (*p* = 0.044) and the LG (*p* = 0.037) in the Anticipated group were significantly greater than those in the Unanticipated group (Table 2).

## 4. Discussion

Our study aimed to explore and compare the neuromuscular control strategies of individuals with chronic ankle instability (CAI) during anticipated and unanticipated landing tasks using non-negative matrix factorization (NNMF) combined with muscle synergy extraction techniques. This study offers the first comprehensive assessment of how CAI patients organize their neuromuscular responses and strategies during unanticipated landings. Our study reveals several key findings: (1) During landing tasks, CAI patients exhibit significant activation of proximal muscles, particularly the gluteus maximus and rectus femoris, demonstrating distinct muscle synergy patterns. (2) In unanticipated landing tasks, CAI patients show an active neuromuscular response with notable adjustments in their ankle and knee control strategies. (3) During unanticipated landings, CAI patients display specific muscle synergy patterns, reflecting how they adapt to unforeseen landing scenarios through various muscle coordination strategies to maintain stability.

Our study reveals that during unanticipated landings, the weight proportions of the gluteus maximus, rectus femoris, and vastus medialis in muscle synergy 1 are significantly higher in individuals with CAI compared to anticipated landings. This finding indicates that proximal joint muscles play a crucial role in managing unanticipated landing tasks in CAI patients [31]. The notable activation of these proximal muscles in such sudden landing situations reflects their central role in maintaining body stability and control [32]. Specifically, our research shows that CAI patients exhibit higher levels of proximal muscle activation during unanticipated landings compared to anticipated landings. This suggests that CAI patients rely on the gluteus maximus, rectus femoris, and vastus medialis to enhance stability and compensate for the compromised ankle function when faced with sudden landing impacts. This phenomenon is consistent with previous studies, which have demonstrated significant biomechanical changes in the proximal hip joint of CAI patients during landings. Ankle joint impairment compels CAI patients to activate hip joint adjustment mechanisms during unanticipated landings to compensate for the functional deficits of the ankle [33,34,35].

Another significant finding of our study is that in synergy 2, the weight of the gluteus maximus and vastus lateralis are notably higher in CAI patients during unanticipated landings compared to anticipated landings, whereas during anticipated landings, the weight of the medial and lateral gastrocnemius is higher. This suggests that CAI patients tend to rely more on proximal knee muscles to enhance body stability during unanticipated landings. This compensatory mechanism likely arises because ankle dysfunction hinders the joint from providing sufficient support during landings, shifting more of the stability task to the proximal muscles around the knee. This compensatory strategy highlights the critical role of proximal muscles in maintaining balance, especially when CAI patients are confronted with sudden, unanticipated landings. The activation of these proximal muscles not only provides support for dynamic balance but also helps absorb the impact forces during landing. Beck et al.’s research further supports this notion, suggesting that activating proximal knee muscles (such as knee flexors) consumes more energy compared to distal muscles (such as ankle plantar flexors), indicating that proximal muscles play a more significant role in absorbing impact and maintaining balance [36]. Furthermore, the importance of the knee in managing landing impacts and maintaining dynamic balance underscores its central role in the compensatory strategies of CAI patients [37]. When ankle function is compromised, the knee becomes a substitute source of power by mobilizing surrounding proximal muscle groups, effectively distributing external forces during landings. This reduces the load on the ankle joint, minimizing the risk of further injury [38,39].

In Synergy 3, the weight of the tibialis anterior (TA) in CAI patients during unanticipated landings is significantly higher compared to anticipated landings. This finding suggests that CAI patients compensate for their lack of ankle stability by increasing the activation of ankle dorsiflexion during unanticipated landings. The enhanced activation of the TA helps stabilize the ankle in a tighter, more secure position, thereby improving overall landing stability. Particularly in response to unanticipated landings, the activation of the TA plays a critical role in controlling the center of mass and preventing imbalance. Gribble’s research on the motor control strategies of CAI patients found that they increase co-activation across major lower limb joints, especially in unstable or dynamic environments, which aligns with our finding regarding the role of the TA during unanticipated landings [40]. The study also highlighted the importance of early TA activation during the initial phase of landing, particularly in terms of ankle co-activation and overall stability. By activating the TA in advance, CAI patients can enhance the support of the ankle joint, better managing unpredictable landing conditions and maintaining dynamic balance. This early response mechanism of the TA not only helps prevent excessive ankle inversion and eversion but also reduces the risk of injury. DeMers’ musculoskeletal modeling study further highlighted that increased contraction of both ankle invertors and evertors enhances overall ankle stability, thereby reducing the risk of potential injuries [41]. By strengthening the use of ankle dorsiflexion and the co-activation mechanism, CAI patients can effectively compensate for their lack of ankle stability, particularly during unanticipated landings, and prevent further damage. Additionally, Kim emphasized the critical role of the TA in compensating for ankle sprain-related deficiencies [42]. Effective activation of the TA enables CAI patients to better control ankle posture and enhance overall stability during dynamic activities. By increasing TA activation, CAI patients can quickly lock the ankle joint during landing, reducing the potential risks associated with ankle instability. This further validates the key role of the TA in maintaining dynamic balance and preventing ankle injuries [43].

Analysis of synergy 4 reveals that the muscle synergy patterns of individuals with CAI during unanticipated landings differ from those observed during anticipated landings. During unanticipated landings, the activation weight of the gluteus maximus and vastus lateralis is significantly higher compared to anticipated landings. This finding indicates that CAI patients rely more on proximal muscles to enhance body stability when faced with sudden landing impacts [34,44]. This compensatory mechanism underscores the critical role of proximal muscles in maintaining stability. Additionally, we observed that during anticipated landings, the activation weights of the medial and lateral gastrocnemius are notably higher than during unanticipated landings. This suggests that CAI patients rely more on the posterior muscle groups of the ankle joint to control and absorb landing impacts during anticipated tasks. The different muscle activation patterns reflect how CAI patients adjust their muscle control strategies to meet the challenges presented by different types of landing tasks. This finding is supported by several researchers. For instance, DeJong et al. noted that CAI patients exhibit significant compensatory muscle activation in proximal joints (such as the knee and hip), indicating that impaired ankle function prompts patients to enhance proximal muscle activity to compensate for ankle deficiencies [45]. This compensatory mechanism is particularly evident during unanticipated landing tasks, where patients rely more on proximal muscles to make up for ankle deficiencies. Kim et al. observed increased activation of knee and hip muscles (such as the vastus lateralis, vastus medialis, gluteus maximus, and gluteus medius) during the transition phase of landing and cutting tasks in CAI patients, which further supports our findings [46]. Rios et al. found that CAI patients demonstrate notably increased activation in proximal muscles during single-leg stance tasks, in contrast to the healthy group, suggesting that CAI patients improve postural control and reduce ankle instability by increasing the activation of proximal muscles [47,48]. In contrast, during anticipated landing tasks, the activation weight of the medial and lateral gastrocnemius muscles is elevated. This phenomenon is consistent with the findings of Gehring et al., who highlighted that in CAI patients, the posterior muscle groups of the ankle (such as the gastrocnemius) play a crucial role in controlling and absorbing landing impacts during anticipated shocks [33,49]. This muscle activation pattern helps patients effectively disperse external forces and reduce the load on the ankle joint during anticipated landings [50]. These findings stress the significance of focusing on proximal muscle function when designing rehabilitation and prevention strategies for CAI patients, while also revealing differences in ankle muscle activation patterns across various landing tasks. Such insights provide a solid foundation for further research and clinical interventions aimed at helping CAI patients improve their motor control strategies, enhance stability, and reduce the risk of injury.

Despite providing valuable insights into the muscle synergy patterns of CAI patients across different landing tasks, this study has several limitations. Notably, the sample size is relatively small, encompassing just 20 CAI patients. A smaller sample size may affect the determination of the optimal number of muscle synergies and could introduce errors in the variance accounted for (VAF), impacting the accuracy of the results. Additionally, a limited sample size may introduce bias during the K-means clustering process, affecting the reliability of the findings. Secondly, this study compared muscle synergy patterns only in CAI patients during anticipated and unanticipated landings, without comparing these patterns to a healthy control group. This limitation restricts our understanding of the differences in muscle synergy between CAI patients and healthy individuals and does not reveal the muscle activation patterns of the healthy group during the same tasks. This comparison is crucial for a deeper understanding of the pathological mechanisms of CAI and the development of effective rehabilitation strategies. Thirdly, the study lacks the recording and interpretation of kinematic and dynamic data. This gap hinders a comprehensive analysis of muscle synergies and their clinical implications. The lack of kinematic and dynamic data limits the exploration of how muscle activation relates to movement performance, thereby affecting our overall understanding of motor control in CAI patients during landings. Finally, the current research primarily focuses on muscle synergy patterns during the landing process, which may be somewhat limited for studies on ankle injury patients. Future research should extend to other movement domains, such as cutting, walking, running, and jumping, to comprehensively assess muscle control strategies under various movement conditions. This approach will help in understanding the characteristics of muscle synergy across different movement patterns.

## 5. Conclusions

This study investigated the changes in muscle synergy patterns in patients with CAI during anticipated and unanticipated landing tasks and the influence of these changes on neuromuscular control strategies. The findings demonstrated that CAI patients exhibit distinct muscle synergy patterns across the two landing conditions, which prompts the adoption of different neuromuscular control strategies to cope with the varying demands of each task. In unanticipated landings, CAI patients rely more heavily on the activation of proximal muscles, such as the gluteus maximus and vastus lateralis. This compensatory activation emphasizes their attempt to bolster hip and knee stability, compensating for the impaired function at the ankle joint. In contrast, during anticipated landings, CAI patients predominantly engage the posterior ankle muscles, particularly the medial and lateral gastrocnemius, to better manage and absorb the expected landing impact. These differences in muscle activation patterns suggest that CAI patients adapt their neuromuscular control strategies in response to the specific challenges posed by different landing tasks. This nuanced understanding of task-dependent muscle synergy adaptations may provide valuable insights for rehabilitation approaches aimed at improving functional stability in CAI patients.

## Figures and Tables

**Table 1:** Descriptive data of the number of muscle synergies, global VAF each group.

	Unant Mean (SD)	Ant Mean (SD)	*p*-Value
The number of muscle synergies Global VAF (%)	4.15(0.62)		
With three synergies	84.93 (1.54)	85.28 (1.51)	0.153
With four synergies	90.03 (0.94)	90.53 (1.19)	0.139
With five synergies	92.4 (0.80)	92.71 (0.89)	0.192
Local VAFs with four synergies (%)			
GM	94.83 (3.23)	89.37 (4.79)	0.105
RF	89.32 (4.23)	88.18 (3.37)	0.661
VM	93.75 (3.10)	91.41 (2.87)	0.327
VL	89.73 (4.16)	88.54 (4.97)	0.504
MG	93.26 (2.01)	95.14 (2.56)	0.411
LG	92.19 (6.35)	96.13 (3.11)	0.226
SL	91.75 (3.01)	92.36 (1.93)	0.723
TA	92.46 (4.58)	92.15 (3.53)	0.869
PL	94.06 (5.03)	90.11 (2.82)	0.202

Note: Unant: Unanticipated group; Ant: Anticipated group; SD: standard deviation.

**Table 2:** Comparison of muscle synergy vectors during landing between the Unanticipated group and Anticipated group.

Synergy Vectors Mean (SD)	Muscle Synergy 1			Muscle Synergy 2			Muscle Synergy 3			Muscle Synergy 4		
	Unant	Ant	*p*-Value	Unant	Ant	*p*-Value	Unant	Ant	*p*-Value	Unant	Ant	*p*-Value
IGM	0.61 (0.22)	0.53 (0.19)	0.041	0.20 (0.12)	0.18 (0.07)	0.679	0.19 (0.09)	0.17 (0.10)	0.733	0.61 (0.25)	0.47 (0.24)	0.038
IRF	0.53 (0.22)	0.47 (0.13)	0.287	0.77 (0.19)	0.73 (0.23)	0.432	0.08 (0.10)	0.10 (0.14)	0.515	0.30 (0.18)	0.28 (0.17)	0.688
IVM	0.35 (0.18)	0.31 (0.10)	0.376	0.55 (0.25)	0.38 (0.20)	0.040	0.16 (0.14)	0.16 (0.15)	0.996	0.77 (0.18)	0.61 (0.15)	0.001
IVL	0.21 (0.13)	0.20 (0.10)	0.953	0.50 (0.16)	0.35 (0.18)	0.014	0.16 (0.10)	0.14 (0.13)	0.583	0.47 (0.12)	0.39 (0.21)	0.140
IMG	0.25 (0.18)	0.22 (0.13)	0.369	0.37 (0.11)	0.41 (0.22)	0.517	0.59 (0.30)	0.71 (0.17)	0.039	0.29 (0.13)	0.42 (0.22)	0.044
ILG	0.22 (0.12)	0.23 (0.11)	0.939	0.30 (0.14)	0.37 (0.19)	0.285	0.46 (0.19)	0.48 (0.17)	0.592	0.30 (0.12)	0.45 (0.16)	0.037
ISL	0.07 (0.08)	0.08 (0.06)	0.808	0.32 (0.19)	0.38 (0.15)	0.397	0.17 (0.04)	0.18 (0.04)	0.974	0.20 (0.10)	0.27 (0.12)	0.123
ITA	0.19 (0.17)	0.21 (0.09)	0.712	0.11 (0.07)	0.14 (0.09)	0.348	0.67 (0.27)	0.51 (0.18)	0.040	0.15 (0.24)	0.13 (0.12)	0.731
IPL	0.11 (0.14)	0.12 (0.10)	0.856	0.33 (0.06)	0.20 (0.10)	0.049	0.27 (0.06)	0.24 (0.05)	0.697	0.43 (0.21)	0.46 (0.26)	0.699

Note: Unant: Unanticipated group; Ant: Anticipated group; SD: standard deviation.

## Data Availability

Data are available on request due to the restriction of ethics.
